# Serological and molecular investigation for brucellosis in swine in selected districts of Uganda

**DOI:** 10.1007/s11250-016-1067-9

**Published:** 2016-05-03

**Authors:** Joseph Erume, Kristina Roesel, Michel M. Dione, Francis Ejobi, Gerald Mboowa, Joseph M. Kungu, Joyce Akol, Danilo Pezo, Hosny El-Adawy, Falk Melzer, Mandy Elschner, Heinrich Neubauer, Delia Grace

**Affiliations:** College of Veterinary Medicine, Animal Resources and Biosecurity, Makerere University, P. O. Box 7062, Kampala, Uganda; International Livestock Research Institute (ILRI), C/O Bioversity International, P. O. Box 24384, Kampala, Uganda; Mycobacteriology (BSL-3) Laboratory, College of Health Sciences, Makerere University, P. O. Box 7072, Kampala, Uganda; Friedrich-Loeffler-Institute, Federal Research Institute for Animal Health, Institute for Bacterial Infections and Zoonoses, Naumburger Str. 96a, 07743 Jena, Germany; International Livestock Research Institute, P. O. Box 30709, Nairobi, Kenya

**Keywords:** Screening, Porcine brucellosis, Yersiniosis, Masaka, Mukono, Kamuli districts

## Abstract

Brucellosis is a notifiable zoonotic disease affecting livestock, humans, and wildlife in Uganda. Pigs can be infected with human pathogenic *Brucella suis* biovars 1 and 3 and can be a significant source of brucellosis for humans. Uganda has a rapidly growing pig population, and the pork consumption per capita is the highest in East Africa. The objective of this work was to determine the seroprevalence of brucellosis in Ugandan pigs. A cross-sectional serosurvey of pigs was conducted in three of the major pig-keeping districts in Uganda (Masaka (*n* = 381 samples), Mukono (*n* = 398), and Kamuli (*n* = 414)). In addition, pigs originating from these districts were sampled in the major pig abattoir in Kampala (*n* = 472). In total, 1665 serum samples were investigated by serological and molecular tests. Only three putative brucellosis-positive samples were detected serologically using indirect ELISA. These sera were found negative for *Brucella* antibodies by CFT; however, two had antibodies against *Yersinia enterocolitica* as determined by SAT. Presence of antibodies against *Yersiniae* was confirmed by *Y. enterocolitica* antibody-specific ELISA. The two *Yersiniae* ELISA-positive samples were brucellosis negative using real-time PCR. We tested additional 142 sera from the 1665 samples with real-time PCR. All tested negative. Under this type of production system, we expect a maximum *B. suis* prevalence of less than 1 % at 95 % confidence level, and therefore, the risk of acquiring brucellosis from the pigs or their products is negligible. However, pigs may harbor the zoonotic *Y. enterocolitica*. This is the first study to investigate the occurrence of brucellosis in pigs in Uganda and the first study to report *Y. enterocolitica* antibodies in swine in Uganda.

## Introduction

Brucellosis is a notifiable zoonotic disease affecting people, livestock, and wildlife globally (Perry and Sones 2007). It is widespread causing significant human suffering and serious economic losses in livestock (Nakavuma and Opuda-Asibo 1999; McDermott et al. 2013). Although national statistics are lacking, there is considerable concern about brucellosis in Uganda. A seroprevalence of 12 and 7 % was reported among beef abattoir workers in Kampala and Mbarara district, respectively (Nabukenya et al. 2013). In Mulago National Refererral Hospital, located in Kampala City, 652 cases of brucellosis were diagnosed between June 2004 and May 2006 alone (Makita et al. 2008).

Brucellosis, caused by *Brucella suis*, is a major disease of pigs causing infertility, production of small litters, and abortion in sows. *B. suis* is a notable occupational hazard particularly to abattoir workers, farmers, and veterinarians (Radostits et al. 2000; Swai and Schoonman 2012). Uganda has reported pig brucellosis to OIE between 1996 and 2000 (Scientific Opinion of the Panel on Animal Health and Welfare AHAW on a request from the Commission on porcine brucellosis Brucella suis 2009), but it is not clear how this was diagnosed and which strains or biovars were involved. Our literature review found no publications on porcine brucellosis in Uganda, but initial scoping studies with farmers revealed high levels of abortion and infertility (Ouma et al. 2014).

Pig production in Uganda can be categorized into three main systems namely (i) intensive, (ii) semi-intensive, and (iii) extensive (small-scale subsistence). Less than 10 % of pigs are kept in intensive or semi-intensive systems but extensive/tethered/small-scale pig-production system is common (Dione et al. 2014). The demand for pork in Uganda is growing rapidly, and the population of pigs has increased from 0.19 million in 1980 to 3.2 million in 2008 (Uganda Bureau of Statistics/Ministry of Agriculture and Fisheries 2009); currently, Uganda has the largest pig population in eastern Africa and ranks 3rd in sub-Saharan Africa (FAOSTAT/FAO 2011). The prevalence of porcine brucellosis in Uganda is unknown though the disease is present in sub-Saharan Africa (Godfroid et al. 2013; Ducrotoy et al. 2015).

The objectives of this work were to determine the seroprevalence of brucellosis in Ugandan pigs, to assess the brucellosis risk from pigs posed to humans, and to characterize the organisms involved. The work was part of Consultative Group for International Agricultural Research (CGIAR) research aiming at characterizing challenges and evaluating options that will allow emerging pig farmers to improve their productivity and livelihoods, while increasing the supply of critical nutrients to their communities and urban centers.

## Methodology

### Study area

The study was conducted from December 2012 to July 2013 in the only official pig abattoir in Kampala City and in pig farms in Masaka, Mukono, and Kamuli districts, Uganda. The three districts studied were selected using a process to identify district representative of high pork production potential (Ouma et al. 2014).

### Abattoir sampling strategy

A cross-sectional abattoir survey was conducted on pigs brought from three districts of Uganda: Masaka, Mukono, and Kamuli. Visits were made twice a week for a period of 3 months when pigs from the target districts were scheduled for slaughter, and blood samples of at least 20 pigs were taken purposively. The blood was collected into sterile vacutainer tubes without anticoagulant, and metadata for each sample included the district of origin and date the animal was sampled. Blood samples were immediately safely transported in a cold box to the College of Veterinary Medicine, Animal Resources and Biosecurity, Makerere University, where sera were harvested and stored at −20 °C until analyzed.

### On-farm sampling strategy

A cross-sectional farm survey was carried out in Masaka, Mukono, and Kamuli districts from April to July 2013. The pig population data from the government livestock census of 2008 was used to facilitate identification of locations within the selected districts where the pig value chain activities would take place. In each district, four to six sub-counties with high pig population were selected for further scrutiny of the existing value chain domains. Three value chain domains were considered: rural production for rural consumption (R-R), rural production for urban consumption (R-U), and urban/periurban production for urban consumption (U-U). In a particular district, two sub-counties were selected to represent each value chain domain. Within each selected sub-county, two to three villages were randomly selected for the pig value chain activities. In total, 35 villages were selected for the project value chain assessment activities (Ouma et al. 2014). For this study and due to financial limitations, 22 out of the 35 villages were purposively selected across the three districts. In the selected villages, a census of all pig farmers was provided by local governmental staff and farmer selection was performed using computer-generated, random numbers. The epidemiological unit was considered to be the farm. In each farm, one animal fitting the inclusion criteria was selected and included in the survey. A structured questionnaire was administered to the owner of the farm. The aim of the questionnaire was to assess the disease risk in the farming system. In each household, the selected pig was restrained and blood was collected from the jugular vein into a sterile vacutainer tube without anticoagulant and serum was harvested immediately upon return from the field.

### Sample size determination

The sample size at each site was calculated considering an infinite population (no recent census data) using the formula adopted from Thrusfield (1995) as follows:

*n* = *Z*^2^*P*(1 − *P*) / *d*^2^, where *n* is the required sample size; *Z* is the multiplier from normal distribution (1.96); *P* is the estimated prevalence which is 50 % considering the fact that there was no reliable prevalence data for brucellosis in Uganda; (1 − P) is the probability of having no disease and *d* is the desired precision (5 %). The level of confidence for the study was set at 95 % confidence interval. Based on this formula, a decision was made to sample a total of 384 farms in each district and was rounded to 400. Additional samples were purposively collected from village boars.

### Criteria for exclusion

Piglets younger than 3 months old, pregnant sows and nursing sows with litters less than 2 months old, as well as weak pigs which might be adversely affected by bleeding were excluded from the survey.

### Laboratory analysis

#### Serological detection of antibodies against *Brucella*

##### Indirect ELISAs

Specific detection of antibodies against *Brucella* in the porcine serum samples was carried out using two indirect ELISA kits: the Ingezim *Brucella* Porcine *Brucella*-Ab indirect ELISA kit (INGENASA, Madrid, Spain) and the ID. Vet Screen® (Grabels, France).

##### Ingezim *Brucella* Porcine *Brucella*-Ab indirect ELISA

A total of 472 serum samples from adult pigs slaughtered at the abattoir and 381 collected on farm from Masaka were screened for antibodies against *Brucella* at Makerere University, College of Veterinary Medicine, Animal Resources and Biosecurity, using Ingezim ELISA according to the manufacturer’s instructions. The Ingezim *Brucella* Porcine *Brucella*-Ab indirect ELISA kit has a sensitivity of 75 %, is highly specific (98 %) (Muñoz et al. 2012), and is based on *Brucella* LPS antigen. The samples were run on *Brucella*-Ab indirect ELISA kit and the optical densities (ODs) were determined in a microplate spectrometer (BioTek EL 800, North Star Scientific, Ltd) at 450-nm wavelength. Positive and negative control serum samples were included in each test. Interpretation of the results was based on S/P calculation: S/P = OD sample/OD positive control. Samples with the S/P higher than or equal to 0.25 were considered as positive, and all those with S/P value less than 0.25 were considered negative.

##### ID. Vet Screen® ELISA

A total of 1193 sera from pigs aged 3 months and older, including village boars, were shipped to Friedrich-Loeffler-Institute (FLI) and screened for antibodies against *Brucella* using indirect ELISA. Of these sera, 381, 398, and 414 were collected from Masaka, Mukono, and Kamuli, respectively. The sera were screened for antibodies against *Brucella* using ID. Vet Screen® (ID. Vet Innovative Diagnostics, Grabels, France) according to the manufacturer’s instructions. ID. Vet Screen® is a robust test that is based on the purified *Brucella* LPS antigen. The results by ID Screen® ELISA were validated by testing the field samples in parallel with archived *Brucella* antibody-positive (14RB6037 FLI, 10RB1462 EUPigBSS) controls, a negative control (13RB4440 FLI), and positive field samples (08RB3522, 08RB3538 FLI) from the 2008 outbreak of brucellosis in wild boars in Germany.

##### Complement fixation test

The complement fixation test (CFT) was performed as a confirmatory test on putative positive samples identified by indirect ELISA. The CFT was carried out at OIE Reference Laboratory for brucellosis (hosted by FLI) following a micromethod according to the Kolmer technique (fixation at 2–8 °C, overnight) and OIE Terrestial Manual 2012 guidelines. The serum samples and positive and negative controls were first diluted 1:5 with CFTB and incubated in a water bath at 60 °C for 30 min to inactivate the complement. Then, in 96-well, U-bottomed microplates, beginning from the second row, 25 μl of inactivated serum samples was serially diluted in CFTB from 1:5 to 1:320, after which 25 μl of brucellosis antigen (IDEXX Pourquier (France) (diluted 1:100 with CFTB)) was added to each well, followed by addition of 25 μl of complement (VIRION, Germany) to each well at working dilution 1:55 in CFTB. Control wells with diluent only, complement and diluent and antigen, and complement titrations 2, 1, 0.5, and 0.25, with diluent and antigen, were prepared to contain 75 μl in each well. The plates were shaken briefly and incubated for 16–20 h in a refrigerator. The plates after overnight incubation were pre-warmed in the incubator at 30 °C for 30 min. Then 50 μl of freshly prepared hemolytic system was added into each well and plates were shaken carefully. The plates were incubated at 37 °C for 15–30 min in a wet chamber. The plates were centrifuged for 5 min at 1000×*g*. The degree of hemolysis was compared with set standards corresponding to 0, 25, 50, 75, and 100 % lysis. The absence of anticomplementary activity was checked for each serum in the first row of the plates. Results were expressed in CFT international units (ICFTU) per millimeter calculated in line with those obtained in parallel titration with national standard serum calibrated against OIE International Standard Serum which contains 595 ICFTU/ml (OIE Terrestial Manual 2012). Sera giving a titer of 20 ICFTU/ml or more were considered to be positive.

##### Slow agglutination test

Slow agglutination to detect agglutinating antibodies against *Yersinia enterocolitica* was performed in U-bottomed microtiter plates as previously described (Burkhardt 1992). The serum samples were serially diluted twofold using 0.005 % solution of safranin in phosphate-buffered saline. Then an equal volume (50 μl) of *Y. enterocolitica* 0:9 standardized antigen (YAG 03/09) prepared from ATCC 55075 reference strain (FLI, Jena, Germany) was added to each well. The plates were shaken on a shaker and incubated in a wet chamber in the incubator at 37 °C for 20–24 h. Test sera were analyzed in parallel with *Brucella*-positive (KS Bruc 03/04) and *Brucella*-negative (KS Bruc 03/04) control sera and *Y. enterocolitica* weak and strong positive sera. After incubation, agglutination titers were determined and expressed as the reciprocal of the final dilution in which agglutination was observed. The results were evaluated as follows: for negative, occurrence of a clear red button/knob of antigen at the bottom; for positive, evaluated according to the degree of agglutination thus (1) film without red center—4+, (2) film with small red center—3+, (3) film with moderate red center—2+, (4) film with intensive/strong red center—1+, and (5) clear red button at bottom—0.

##### Indirect ELISA for *Y. enterocolitica* antibodies

Indirect ELISA using PIGTYPE® YOPSCREEN test kit (Qiagen® GmbH, Hilden, Germany) was used to detect antibodies to pathogenic *Yersiniae* in the pig sera that were positive by SAT. The test was performed according to the manufacturer’s instructions. The results were validated by testing the Uganda samples in parallel with archived *Y. enterocolitica* antibody-positive field samples (13RB4901 FLI, 13RB4902 FLI, and 13RB4903FLI).

#### Molecular detection of *Brucella*

##### DNA preparation from sera

DNA was extracted and purified from porcine serum using DNeasy Blood and Tissue Kit (QIAGEN, Germany) according to the instructions of the manufacturer using QIAcube. The DNA concentration was determined photometrically using a Nano Drop ND-1000 UV-vis spectrophotometer (Nano-Drop Technologies, Wilmington, USA).

##### Real-time PCR

Multiplex real-time PCR was used for detection of the genus-specific *Brucella* cell surface salt extractable bcsp31 kDa protein gene, *Brucella abortus* alkB gene and *Brucella melitensis* BMEI1162 gene from extracted DNA (Probert et al. 2004). The *B. abortus* and *B. melitensis* TaqMan probes target the alkB and BMEI1162 gene, respectively. PCR reaction was performed using primer and probe set is shown in Table 1 (Jena Bioscience GmbH, Germany). The 25 μl multiplex PCR mixture consisted of 2× TaqMan™ Environmental master mix (Applied Biosystems, NJ USA), 200 nM of each primer, 100 nM of each probe, and 5 μl of template DNA. Amplification and real-time fluorescence detection was performed on a Mx3000P thermocycler system (Stratagene, La Jolla, Canada) using the following reaction conditions: 2 min denaturation at 50 °C, polymerase activation step at 95 °C for 10 min, followed by 50 cycles of 95 °C for 25 s and 57 °C for 1 min. The samples scored positive were confirmed by visual inspection of the graphical plots showing cycle numbers versus fluorescence values. A sample with a fluorescence signal 30 times greater than the mean standard deviation in all wells over cycles 2 through 10 was considered a positive result, whereas a sample yielding a fluorescence signal less than this threshold value was considered a negative result. Cycle threshold values below 40 cycles were interpreted as positive.

**Table 1:** Oligonucleotide primers and probes used in the real-time multiplex PCR assay for the detection of *Brucella* spp., *B. abortus*, and *B. melitensis*

Target	Primer	
*Brucella* spp.	5′GCTCGGTTGCCAATATCAATGC 3′	Forward
	5′GGGTAAAGCGTCGCCAGAAG 3′	Reverse
	FAM-AAATCTTCCACCTTGCCCTTGCCATCA-BHQ1	Probe
*B. abortus*	5′GCGGCTTTTCTATCACGGTATTC 3′	Forward
	5′CATGCGCTATGATCTGGTTACG 3′	Reverse
	HEX-CGCTCATGCTCGCCAGACTTCAATG-BHQ1	Probe
*B. melitensis*	5′AACAAGCGGCACCCCTAAAA 3′	Forward
	5′CATGCGCTATGATCTGGTTACG 3′	Reverse
	CY5-CAGGAGTGTTTCGGCTCAGAATAATCCACA-BHQ2	Probe

*FAM* carboxyfluorescein, *HEX* hexachlorofluorescein, *BHQ1* Black Hole Quencher 1, *BHQ2* Black Hole Quencher 2

Since age has been alluded to as a significant risk factor for bovine brucellosis (Muñoz et al. 2012), we performed real-time PCR on almost all (86.57 %, *n* = 58) “village boar” samples. Additionally, ELISA positive and other sera, amounting to 12 % of 1193 on farm serum samples, were tested by real-time PCR.

### Data analysis

Standard statistical analyses were performed using Microsoft Office Excel 2010. The disease prevalence was calculated by dividing the number of positive samples by the total number of samples tested. Test sensitivity (Se) of 75 % and specificity (Sp) of 98 % for i-ELISA (Muñoz et al. 2012) were used to calculate apparent prevalence of antibodies against *Brucella* infection according to Greiner and Gardner (2000).

## Results

### Abattoir seroprevalence of porcine brucellosis

Of the 472 serum samples tested, 332, 2, and 138 sera were collected from pigs originating from Masaka, Mukono, and Kamuli districts, respectively. None of the serum samples was positive for *Brucella* antibodies, indicating 0 % apparent abattoir prevalence (95 % CI: 0.008) of porcine brucellosis in the three districts, suggesting absence of active *Brucella* exposure.

### On-farm seroprevalence of porcine brucellosis

A total of 1 (0.28 %), 0 (0 %), and 1 (0.25 %) serum samples were putatively seropositive for *Brucella* in Masaka, Mukono, and Kamuli districts, respectively (Table 2). The positive sample from Masaka was detected by Ingezim ELISA and that from Kamuli by ID. Vet Screen®. There was no agreement between the results of the two tests on Masaka samples. The Ingezim ELISA kit was not employed for testing samples from Mukono and Kamuli districts.

**Table 2:** Seroprevalence of porcine brucellosis in Masaka, Mukono, and Kamuli districts as detected by indirect ELISA

District	Village	Sub-county	Number samples^a^	Number brucellosis positive^b^	
				Makerere (Ingezim porcine iELISA)	FLI (ID. Vet Screen iELISA)
Masaka	Kisoso	Kkingo	43 (2)	1	0
	Ssenya	Kkingo	34 (0)	0	0
	Lukindu	Kyanamukaka	37 (0)	0	0
	Kanoni-Bukunda	Kyanamukaka	50 (1)	0	0
	Senyange A	Ivan Kakembo	50 (0)	0	0
	Kyamuyimbwa Kikalala	Kabonera	35 (0)	0	0
	Butego	Katwe-Butego	27 (0)	0	0
	Kijjabwemi	Kimanya-Kyabakuza	43 (0)	0	0
	Kyabakuza B	Kimanya-Kyabakuza	35 (0)	0	0
Mukono	Kazo/Kalagala	Tenjeru	52 (1)	Nd	0
	Nsanja/Gonve	Tenjeru	43 (0)	Nd	0
	Bugoye/Kabira	Tenjeru	48 (0)	Nd	0
	Kyoga	Kyampisi	52 (0)	Nd	0
	Dundu	Kyampisi	61 (0)	Nd	0
	Kitete	Mukono TC	55 (0)	Nd	0
	Joggo	Mukono TC	64 (0)	Nd	0
Kamuli	Balubweneiwa	Bugulumbya	46 (0)	Nd	0
	Bukyonza B	Bugulumbya	30 (0)	Nd	0
	Butabala	Kitayunjwa	40 (0)	Nd	0
	Isingo A	Namwendwa	70 (0)	Nd	0
	Ntansi	Butansi	105 (0)	Nd	1
	Kantu zone	Butansi	110 (0)	Nd	0
Total	22		1130 (4)	1	1

*Nd* not done

^**a**^In brackets are the number of village boars sampled from each village as part of random sample

^b^Sites where samples were tested

### Brucellosis status among purposively sampled village boars

Overall, 63 samples from purposively sampled village boars were analyzed; of these, 27, 25, and 13 were from Masaka, Mukono, and Kamuli, respectively. In total, 0 (0.0 %), 0 (0 %), and 1 (7.69 %) serum samples were putatively seropositive for *Brucella* in Masaka, Mukono, and Kamuli districts, respectively, as detected by ID. Vet Screen® (Table 3).

**Table 3:** Porcine brucellosis in purposively sampled village boars from Masaka, Mukono, and Kamuli districts as detected by indirect ELISA

District	Village	Sub-county	Number of village boar samples	Number brucellosis positive^a^	
				Makerere (Ingezim porcine iELISA)	FLI (ID. Vet Screen iELISA)
Masaka	Kisoso	Kkingo	2	Nd	0
	Ssenya	Kkingo	5	Nd	0
	Lukindu	Kyanamukaka	1	Nd	0
	Kanoni-Bukunda	Kyanamukaka	4	Nd	0
	Senyange A	Ivan Kakembo	3	Nd	0
	Kyamuyimbwa Kikalala	Kabonera	8	Nd	0
	Butego	Katwe-Butego	1	Nd	0
	Kijjabwemi	Kimanya-Kyabakuza	2	Nd	0
	Kyabakuza B	Kimanya-Kyabakuza	1	Nd	0
Mukono	Kazo/Kalagala	Tenjeru	8	Nd	0
	Nsanja/Gonve	Tenjeru	4	Nd	0
	Bugoye/Kabira	Tenjeru	0	Nd	0
	Kyoga	Kyampisi	2	Nd	0
	Dundu	Kyampisi	2	Nd	0
	Kitete	Mukono TC	3	Nd	0
	Joggo	Mukono TC	4	Nd	0
Kamuli	Balubweneiwa	Bugulumbya	2	Nd	0
	Bukyonza B	Bugulumbya	1	Nd	0
	Butabala	Kitayunjwa	1	Nd	1
	Isingo A	Namwendwa	0	Nd	0
	Ntansi	Butansi	9	Nd	0
	Kantu zone	Butansi	0	Nd	0
Total	22		63		1

*Nd* not done

^a^Sites where samples were tested

### Testing of samples with complement fixation test

For confirmation, the two samples that presumptively tested positive by indirect ELISA (ID. Vet Screen®) were subjected to CFT analysis at FLI. Sera were tested in parallel with FLI *Brucella* antibody strong positive (14RB7378) and weak positive (14RB7379) samples. Both putatively positive samples from Ugandan swine tested negative for *Brucella* antibodies by CFT.

### Ugandan pigs positive for antibodies against *Y. enterocolitica*

To check if the detected antibodies were due to cross-reactivity to *Y. enterocoltica*, the two putative positive samples were subjected to slow agglutination test for *Y. enterocolitica* 0:9 antibodies. Both samples from Uganda (14RB7097 and 14RB7324) tested positive for *Y. enterocolitica* antibodies by slow agglutination test, with titers of 40/2 and 40/3, respectively, indicating a 0.17 % prevalence of *Y. enterocolitica* antibodies in the pigs tested. To confirm their serological status, these samples were tested for *Y. enterocolitica* antibodies by indirect ELISA using PIGTYPE® YOPSCREEN kit (Qiagen® GmbH, Hilden, Germany). One sample (14RB7097) tested positive while the other (14RB7324) was negative.

### Testing of serum samples with real-time PCR

To confirm the absence of *Brucella* infection, the *Y. enterocolitica*-positive sample and 143 other sera from Uganda [that comprised of 58 samples from boars (86.57 % of all boars), and 45 and 40 randomly selected sera that showed higher and low ODs in indirect ELISA, respectively, representing 12.07 % (*n* = 144) of the total serum samples] were tested for brucellosis using real-time PCR. The other *Y. enterocolitica*-positive sample could not be tested by real-time PCR due to lack of serum. No *Brucella*-specific DNA signal was detected in all samples tested.

### Statistical analysis

Considering sample size to detect disease for infinite population: *n* = *ln*(*alpha*) / *ln*(*1* − *p*), where *p =* the minimum prevalence, and if assuming the *p* to be 5 % then *n = ln*(*0.05*) / *ln*(*0.95*) = 58.4. This work analyzed 1130 randomly collected samples, so reversing the equation *ln* (*alpha*)/*n* = *ln*(1 − *p*) gives

$$ \begin{array}{c}\hfill ln(alpha)/n = ln\left(1-p\right)\hfill \\ {}\hfill ln\left(1-p\right)= ln(0.05)/1130 = -0.00265\hfill \\ {}\hfill p = 1 - \left( exp\left(-0.00265\right)\right) = 1-0.9973 = 0.0027.\hfill \end{array} $$

Therefore, the sample size used in this study was sufficient to find the disease if it was present in 0.27 % of the population.

Assuming a perfect test (Se = 1), the prevalence in the population must be maximum of 0.27 % for us not to have found it in 1130 samples. The Ingezim Brucella Porcine Ab i-ELISA shows a sensitivity of 75 %; therefore, the prevalence will be no higher than 0.81 %.

## Discussion

To our knowledge, this is the first study to investigate the occurrence of brucellosis in pigs in Uganda or other East African countries. None of the sera tested was positive for porcine brucellosis. Under this type of production system, we expect a maximum *B. suis* prevalence of less than 1 % at 95 % confidence level. Since this study included pigs from Mukono and Masaka, two of the districts with the highest density of pigs in Uganda, and failed to detect disease there, it is likely to be absent.

Our findings that *Brucella* antibodies were absent from a large and representative sample of pigs in the major pig-producing districts of Uganda are consistent with earlier reports of 0 % (Cadmus et al. 2006) and 0.6 % (Onunkwo et al. 2011; Nwanta et al. 2011) prevalence of porcine brucellosis in Nigeria. A 0 % prevalence was also found in commercial piggeries in Zambia (Stafford et al. 1992). Our results however differed from those of Ngbede et al. (2013) who reported a high seroprevalence of porcine brucellosis (30.6 %) in Benue State, Nigeria. All the serological studies reporting the occurrence of porcine brucellosis in Nigeria used RBT for testing whereas our study employed multiple tests. RBT reportedly has high sensitivity (100 %) but is less specific (84 %) (Akhtar et al. 2010).

The finding of 0.17 % prevalence of *Y. enterocolitica* antibodies in our study population is of public health significance. *Y. enterocolitica* is an important zoonotic agent of global concern (Bhaduri et al. 2005; Ifeoma 2013) that can cause serious human suffering ranging from fever, abdominal pain, diarrhea to more serious complications such as appendicitis and erythema nodosum (Bari et al. 2011). Pigs are reported to be the main reservoir of these organisms (Adesiyun and Krishnan 1995; Bhaduri et al. 2005; Fredriksson-Ahomaa et al. 2010). To the best of our knowledge, this is the first report on the occurrence of *Y. enterocolitica* exposure in Ugandan pigs and calls for research on the infection and risks associated with this pathogen. Further studies are needed to investigate the prevalence and impact of *Y. enterocolitica* on Ugandan pigs and to investigate other causes for the high reported levels of abortion and infertility in pigs.

## References

[CR1] Adesiyun AA, Krishnan C (1995). Occurrence of *Yersinia enterocolitica* 0:3, *Listeria monocytogenes* 0:4 and thermophilic *Campylobacter* spp. in slaughter pigs and carcasses in Trinidad. Food Microbiology.

[CR2] Akhtar, R., Chaudhry, Z.I., Shakoori, A.R., Mansoor ud Din, A. and Aslam, A., 2010. Comparative efficacy of conventional diagnostic methods and evaluation of polymerase chain reaction for the diagnosis of bovine brucellosis. Veterinary World, 3(2), 53--56

[CR3] Bari L, Hossain MA, Isshiki K, Ukuku DO (2011). Behavior of *Yersinia enterocolitica* in Foods. Journal of Pathogens.

[CR4] Bhaduri S, Wesley IV, Bush EJ (2005). Prevalence of Pathogenic *Yersinia enterocolitica* Strains in Pigs in the United States. Applied and Environmental Microbiology.

[CR5] Burkhardt, F., 1992. Mikrobiologische Diagnostik(Georg Thieme Verlag Stuttgart–New York)

[CR6] Cadmus SIB, Ijagbone IF, Oputa HE, Adesokan HK, Stack JA (2006). Serological survey of Brucellosis in Livestock Animals and Workers in Ibadan, Nigeria. African Journal of Biomedical Research.

[CR7] Dione MM, Ouma EA, Roesel K, Kungu J, Lule P, Pezo D (2014). Participatory assessment of animal health and husbandry practices in smallholder pig production systems in three high poverty districts in Uganda. Preventive Veterinary Medicine.

[CR8] Ducrotoy, M., Bertu, W.J., Matope, G., Cadmus, S., Conde-Álvarez, R., A.M. Gusi, A.M., Welburn, S., Ocholi, R., Blasco, J.M. and Moriyón, I., 2015. Brucellosis in Sub-Saharan Africa: Current challenges for management, diagnosis and control. Acta Tropica. doi:10.1016/j.actatropica.2015.10.023 10.1016/j.actatropica.2015.10.023 26551794

[CR9] FAOSTAT/FAO, 2011. Food Balance/Food Supply - Livestock and Fish Primary Equivalent. Retrieved January 5, 2016, from http://faostat3.fao.org/browse/FB/CL/E.

[CR10] Fredriksson-Ahomaa M, Meyer C, Bonke R, Stüber E, Wacheck S (2010). Characterization of *Yersinia enterocolitica* 4/O:3 isolates from tonsils of Bavarian slaughter pigs. Letters in Applied Microbiology.

[CR11] Godfroid J, Al Dahouk S, Pappas G, Roth F, Matope G, Muma J, Marcotty T, Pfeiffer D, Skjerve E (2013). A "One Health" surveillance and control of brucellosis in developing countries: moving away from improvisation. Comparative Immunology, Microbiology and Infectious Diseases.

[CR12] Greiner M, Gardner IA (2000). Application of diagnostic tests in veterinary epidemiologic studies. Preventive Veterinary Medicine.

[CR13] Ifeoma MM (2013). Incidence and Public Health Significance of *Yersinia enterocolitica* in Enugu Urban-Enugu State, Nigeria. Journal Of Environmental Science, Toxicology And Food Technology.

[CR14] Makita K, Fèvre EM, Waiswa C, Kaboyo W, De Clare Bronsvoort BM, Eisler MC, Welburn SC (2008). Human brucellosis in urban and peri-urban areas of Kampala, Uganda. Annals of the New York Academy of Sciences.

[CR15] McDermott J, Grace D, Zinsstag J (2013). Economics of brucellosis impact and control in low-income countries. Revue scientifique et techniquecpcOffice International des Epizooties.

[CR16] Muñoz PM, Blasco JM, Engel B, de Miguel MJ, Marín CM, Dieste L, Mainar-Jaime RC (2012). Assessment of performance of selected serological tests for diagnosing brucellosis in pigs. Veterinary Immunology and Immunopathology.

[CR17] Nabukenya I, Kaddu-Mulindwa D, Nasinyama GW (2013). Survey of *Brucella* infection and malaria among Abattoir workers in Kampala and Mbarara Districts. Uganda. BMC Public Health..

[CR18] Nakavuma JK, Opuda-Asibo J (1999). Sero study of *Brucella abortus* in cattle and goat in central and southern Uganda. Uganda Journal of Agricultural Sciences.

[CR19] Ngbede EO, Momoh AH, Ruben Sylvester Bala RS, Madaki BD, Maurice NA (2013). An Abattoir-Based Study on Serodiagnosis of Swine Brucellosis in Makurdi, Benue State, North-Central Nigeria. Journal of Advanced Veterinary Research.

[CR20] Nwanta JA, Shoyinka SVO, Chah KF, Onunkwo JI, Onyenwe IW, Eze JI, Iheagwam CN, Njoga EO, Onyema I, Ogbu KI, Mbegu EC, Nnadozie PN, Ibe EC, Oladimeji KT (2011). Production characteristics, disease prevalence, and herd-health management of pigs in Southeast Nigeria. Journal of Swine Health and Production.

[CR21] Onunkwo JI, Njoga EO, Nwanta JA, Shoyinka SVO, Onyenwe IW, Eze JI (2011). Serological Survey of Porcine *Brucella* Infection in South East, Nigeria. Nigerian Veterinary Journal.

[CR22] Ouma, E., Dione, M., Lule, P., Roesel, K. and Pezo, D., 2014: Characterization of smallholder pig production systems in Uganda: constraints and opportunities for engaging with market systems. Livestock Research for Rural Development, 26(3), Article #56. Retrieved January 5, 2016, from http://www.lrrd.org/lrrd26/3/ouma26056.htm

[CR23] Perry, B. and Sones, K., 2007. Poverty reduction through animal health. Science, 315 (5810), 333—334, doi: 10.1126/science.1138614 10.1126/science.1138614 17234933

[CR24] Probert WS, Schrader KN, Khuong NY, Bystrom SL, Graves MH (2004). Real-time Multiplex PCR Assay for Detection of *Brucella* spp., *B. abortus*, and *B. melitensis*. Journal of Clinical Microbiology.

[CR25] Radostits OM, Gay CC, Blood DC, Hinchcliff KW (2000). Veterinary Medicine: A textbook of the diseases of cattle, sheep, pigs, goats and horses.

[CR26] Scientific Opinion of the Panel on Animal Health and Welfare (AHAW) on a request from the Commission on porcine brucellosis (*Brucella suis*)., 2009. The EFSA Journal, 1144, 1--112. Retrieved January 5, 2016 from http://www.efsa.europa.eu/sites/default/files/scientific_output/files/main_documents/1144.pdf

[CR27] Stafford K, Tafford Y, Paton D, Gamble P (1992). Antibodies to some swine disease in commercial piggeries in Central Zambia. Revue d‘élevage et de médecine vétérinaire des pays tropicaux.

[CR28] Swai ES, Schoonman L (2012). A survey of zoonotic diseases in trade cattle slaughtered at Tanga city abattoir: a cause of public health concern. Asian Pacific Journal of Tropical Biomedicine.

[CR29] OIE Terrestial Manual, 2012. Chapter 2.4.3. Bovine brucellosis, 616--650

[CR30] Thrusfield M (1995). Veterinary Epidemiology.

[CR31] Uganda Bureau of Statistics/Ministry of Agriculture, Animal Industry & Fisheries, 2009. A summary report of the livestock census, 2008.

